# Supplementary material for: 25-Hydroxycholesterol 3-Sulfate Recovers Acetaminophen Induced Acute Liver Injury via Stabilizing Mitochondria in Mouse Models
Source: Cells. 2021 Nov 5;10(11):3027. doi: 10.3390/cells10113027 (PMC8616185; doi:10.3390/cells10113027)
Supplement: Supplementary file 1 [file cells-10-03027-s001.zip › cells-1422419-supplementary.pdf]

Table S1. Primer Sequence for Real-time Polymerase Chain Reaction

Gene name	Gene Accession ID	Forward primer	Reverse primer
Fgf11	NM_010198	TAGCCTGATCCGACAGAAGC	GGCAGAACAGTTTGGTGACG
Pik3cb	NM_029094	CTATGGCAGACAACCTTGACAT	CAGAAGGAAATCGACGGATATGG
Mapk1	NM_001038663	GGTTGTTCCCAAATGCTGACT	CAACTTCAATCCTCTTGTGAGGG
Pdgfa	NM_008808	GAGGAAGCCGAGATACCCC	TGCTGTGGATCTGACTTCGAG
Pdgfb	NM_011057	ATTGTGCGAAAGAAGCCCATC	GGGTCACTACTGTCTCACACTT
Ywhaz	NM_001253806	TGAGCTGTCGAATGAGGAGAG	CCTCCACGATGACCTACGG
Tlr4	NM_021297	AAATGCACTGAGCTTTAGTGGT	TGGCACTCATAATGATGGCAC
Map3k6	NM_016693	CGGCCTCTCAGTGTGGTCTA	CCAGCGTCGCAAAGGGTAG
Tgfbr1	NM_009370	ATATCTGCCATAACCGCACTG	CTGAAATGAAAGGGCGATCTAGT
Map4k4	NM_001252200	GCACATCTCCATATTCACCACG	GCGCTTACACCAAAATCAACAAG
Rn18s	NR_003278	AGGGGAGAGCGGGTAAGAGA	GGACAGGACTAGGCGGAACA

Table S 2. Summary of The Whole Genome Bisulfite Sequencing Data

Sample name	Raw_reads	clean_reads	Clean_ratio(%)	Uniquely mapped reads	Uniquely mapped rate (%)	sites_covgMean	sites_numCovg10	BS conversion rate(%)
Vehicle	310,214,493	293,764,421	84.68	226,110,474	76.79	19.85	84.79	99.495
25HC3S	322,451,254	305,610,412	84.81	233,822,526	76.51	20.48	85.29	99.496

Table S 3. Significant Enrichment KEGG Pathways of hypomethylated DMGs in Promoter Region under CG context

Pathway name	P-value	Pathway name	P-value	Pathway name	P-value
Prostate cancer	0.001957	MAPK signaling pathway	0.017418	Focal adhesion	0.041224
Cell cycle	0.001957	Colorectal cancer	0.023097	Ubiquitin mediated proteolysis	0.041224
Melanoma	0.00279	Pancreatic cancer	0.024591	Alcoholism	0.041224
Phospholipase D signaling pathway	0.00279	Glutamatergic synapse	0.029023	Type II diabetes mellitus	0.043909
PI3K-Akt signaling pathway	0.002967	Thyroid hormone signaling pathway	0.029938	mRNA surveillance pathway	0.044004
Rap1 signaling pathway	0.004439	Chronic myeloid leukemia	0.030412	Adrenergic signaling in cardiomyocytes	0.04559
Regulation of actin cytoskeleton	0.004439	Adherens junction	0.030412	Endometrial cancer	0.04559
Axon guidance	0.004699	Ras signaling pathway	0.030412	Hippo signaling pathway	0.045993
Endocrine resistance	0.004699	Platelet activation	0.031776	Lysine degradation	0.045993
Pathways in cancer	0.004699	Sphingolipid signaling pathway	0.03248	Toll-like receptor signaling pathway	0.046911
Endocytosis	0.005013	Salmonella infection	0.03248	AGE-RAGE signaling pathway in diabetic complications	0.049037
Choline metabolism in cancer	0.005013	Vascular smooth muscle contraction	0.033906	Chagas disease (American trypanosomiasis)	0.049089
Glioma	0.005013	FoxO signaling pathway	0.039573	Non-small cell lung cancer	0.049089
Hepatitis B	0.005017	Small cell lung cancer	0.039573		
Oocyte meiosis	0.008398	Gap junction	0.039632		
Hedgehog signaling pathway	0.009069	Hepatitis C	0.039632		
EGFR tyrosine kinase inhibitor resistance	0.010398	Signaling pathways regulating pluripotency of stem cells	0.04119		
ErbB signaling pathway	0.012624	Chemokine signaling pathway	0.04119		
HTLV-I infection	0.013355	Progesterone-mediated oocyte maturation	0.04119		
Viral carcinogenesis	0.014992	Insulin signaling pathway	0.04119		
Glycerophospholipid metabolism	0.014992	Cytokine-cytokine receptor interaction	0.041224		
